# Hospital and Institutional News

**Published:** 1913-02-15

**Authors:** 


### HOSPITAL AND INSTITUTIONAL NEWS.

### THE HOSPITAL SATURDAY FUND DINNER.

At the annual dinner of the Hospital Saturday Fund on February 8 there was some excellently plain speaking when the toasts of the evening were proposed and responded to. Some of the speakers emphasised very forcibly the point of view of the ordinary hospital patient of the wage-earning class, who finds himself mulcted every week by an Act he was never consulted about and would certainly have voted against if he had had the chance. This intelligent and respectable citizen now finds himself unable to obtain from hospitals some of those forms of medical treatment for slight injuries and diseases which he has been accustomed to receive, and is somewhat annoyed when requested to obtain his requirements from a panel doctor, a person for whose attainments and standards of conduct he has very often a somewhat scant respect. Accordingly he grumbles when the annual appeal for a small donation to the Hospital Saturday Fund is pressed upon him by the workers who voluntarily act as collectors for that admirable organisation. And the outcome of his dissatisfaction is a reduction of £2,000 in the year's takings of the Fund, and broad hints of an even greater shortage this coming year.

### THE OTHER SIDE OF THE SHIELD.

Other speakers endeavoured to show the point of view of hospital managers, equally unconsulted when the Insurance Act was in preparation. They find themselves with a diminished subscription list (due to the Act), and with an increase of taxation as employers of labour. (In passing, their employees for the most part get no benefit out of the Act which they did not previously enjoy.) They see the State undertaking to provide the insured persons with medical attendance for ordinary ailments; and they find their honorary medical staff disinclined to do for nothing the very work which their brethren on the panel are being paid to do. They know that their proper function is to provide the wage-earners with such kinds of medical aid as the latter are unable to provide for themselves; and they feel themselves morally unjustified in using charitable subscriptions and endowments for those with whom the panel practitioners are quite competent to deal. They see a chance of resuming their original functions, and of avoiding financial ruin at the same time, by referring to panel doctors all cases of minor ill-health in insured persons. The two points of view were well represented at the Saturday Fund dinner, and the upshot of the speeches will surely be that each side will be able to appreciate better the case as it presents itself to the other. Such mutual understanding is bound to create mutual sympathy, and that atmosphere of conciliation, conference, and compromise for which the Hon. Harry Lawson pleaded.

### THE INFIRMARY CHAPEL SUIT.

On February 5 Lord Justice Farwell pronounced judgment in the action brought by Messrs. W. Sutton, W. Sutton, jun., R. Mayne, and E. R. Newbigin against the Remaining Governors of the Royal Victoria Infirmary for the Sick and Lame Poor of the Counties of Newcastle-on-Tyne, Northumberland, and Durham. Our readers will remember that this litigation has been in train for a long time, and that the religious feuds which started it date back to the opening of the new buildings of the infirmary in September 1906, and to the consecration by the Bishop of Newcastle of the chapel which formed part of the reconstructed infirmary. The plaintiffs prayed for a declaration by the Court that, notwithstanding the dedication service, whose effect was to allow Church of England ceremonies alone to take place in the chapel, the trustees should be directed to permit the chapel and the site of the chapel to be used for the general purposes of the charity; in which they desired to include religious services whether of the Church of England or of any other denomination. We shall not now enter further into the details of this deplorable quarrel, except to say that both sides were partly in the right and partly in the wrong. Lord Justice Farwell's judgment states the unprejudiced view of the case with admirable fairness and clearness; and it is quite refreshing to find common sense and the law so conspicuously identified as in his review of the case. He made it clear that, in his opinion, the consecration should not have taken place, and that those of the governors who arranged for it were acting quite *ultra vires*. Any objection entered then would apparently have been upheld by his lordship; but once the deed was done he found it impossible to annul it, and, therefore, the suit of the plaintiffs' failed. Thus the chapel remains exclusively for Church of England services.

### HOW TO SETTLE THE QUARREL.

The judgment and wise summing up of Lord Justice Farwell in the above case contain points due reflection upon which will eventually lead, we hope, to a settling of the long-drawn-out dispute. The house committee are absolved from the suspicion of sharp practice and reactionary procedure. The example of the military authorities, which we have before referred to under this heading, is praised. They very prudently leave religious buildings unconsecrated so as to exclude sectarian bickerings, and avoid the waste of money consequent upon an attempt to provide different places of worship for all the different varieties of creeds. Hospital authorities will do well to take note; and one need not take legal opinion to become aware that "practical difficulties" may well be expected when "land consecrated is consecrated for ever." Such finality verges on the anachronistic; it is hardly to be reconciled with the spirit of our day. Accordingly, it would be well for the victors in this action to yield a little in their turn. Some priority and privilege might be reserved for Church of England services, as a recognition both of the position of an established Church and also of the generosity of those Anglican Churchmen who contributed to the equipment of this particular hospital chapel. But it would seem inequitable and imprudent to exclude Nonconformists altogether, and thus offend a large section of the charity's governors. In the end, the bitter end (for we have Aristotle's authority for it that nothing divides men like differences in religion and in wealth), such a course could only lead to a reconstitution of the executive body, with resulting loss of the services of valuable and experienced institutional workers. To build a separate place of worship for Nonconformists we hold to be, in the present state of hospital finance, economically unjustifiable: it would, too, be rejected as a solution by that agnostic opinion, clear signs of which among parties interested will have been observed by close students of this long dispute, and which is often associated with democratic control. Even in small matters like the present one, it is unwise, in the year 1913, to pursue a clerico-aristocratic policy, especially on turbulent Tyneside.

### THE SAGA OF SCOTT.

Face to face with the glorious tragedy of the Scott expedition, the first feeling is naturally one of melancholy regrets. After that comes an uplifting of spirit at the thought of the honour and privilege that is ours of being fellow-countrymen of the illustrious explorer and his party. Perhaps as yet it were well to be content with such feelings of pity, admiration, and exultation for the lost band of heroes and their deeds. But we cannot forget that one of the gallant five whom we mourn was Dr. E. A. Wilson, the chief of the scientific staff of the expedition. Dr. Wilson was a Cambridge and St. George's man. He was a member of Scott's previous expedition and was one of the sledging party which accomplished the then furthest south. Dr. Wilson was an accomplished scientist in many departments, a sanitarian and medical officer of the highest class, an amateur artist in water-colours of quite exceptional talent, and a strenuous worker and explorer for whom no day was ever too long and no labour too great. At the time of his first expedition twelve years ago Dr. Wilson was, we believe, recovering from an attack of tuberculosis of the lungs. The life of an Antarctic explorer would seem to exercise a beneficent effect upon this condition, for Wilson was specially selected by Scott in 1901 for his furthest south party. Thus the Navy, the Army, the medical profession, and, in a less intimate degree, the London institutional world, are joined together in a common grief, exceeding in a measure that universal sorrow which the whole civilised world is expressing to-day.

### NURSES AND THE INSURANCE ACT.

Among the many unremedied defects and injustices of the Insurance Act, one stands out prominent. It is the effect of the Act and the regulations under the Act upon the nursing profession, and especially the nursing staff of hospitals, voluntary and rate-supported. So far nothing has come of the promises that something would be done to mitigate the hardships which the Act inflicts upon a class more deserving of consideration than most of those which come within the scope of the Act. Mr. Paterson, who took up the cudgels for the nurses when the Act was still a Bill, now returns to the charge. The wise and temperate letter in which he presses the claims of the nursing profession to justice and fair treatment appeared in Tuesday's *Times*, and will appeal in no small degree to all who are interested in institutional affairs, as well as to nurses themselves. It is especially to be noted that in June 1911 Mr. Paterson was given to understand unofficially that the substance of his plea for the nurses would be recognised by amendments on the Report stage of the Bill. This was never done, and it adds even greater force to the arguments in favour of tardy reparation now. We cannot imagine that anyone, of whatever political persuasion he may be, can read this letter without admitting that injustice has been committed, and without desiring to have it undone somehow. The whole problem of medical benefits for nurses will be dealt with in our National Insurance Department next week; and some practical suggestions for solving an anomaly and terminating a gross public fraud will be put forward. These suggestions presuppose that Insurance Committees are willing to approach the matter in a reasonable spirit. From one of the facts which we shall have to quote it would seem that this cannot absolutely be relied upon, another proof—if proof were wanted—of the folly of passing undebated Bills and then administering them in the supposed interests of a political party instead of in those of the people.

### THE LONDON HOSPITAL QUINQUENNIAL APPEAL.

The present year brings round again the special appeal which is made on behalf of the London Hospital every five years; and, as usual, it is being prosecuted with the energy and skill which have made it so successful in previous quinquennia. The task which the managers are setting themselves is the raising of £100,000 extra beyond the £110,000 which is needed each ordinary year to keep the hospital going. The sum is a large one, but experience shows that the London Hospital has many friends, and that there are plenty of potential subscribers who can be got at by personal effort on the part of those interested in the welfare of the hospital. Everything, too, that adroit advertisement can do for the appeal is being done. Thus a few days ago *The Times* contained a full-page advertisement, cleverly displayed so as to catch and hold attention, of the merits of the work which the hospital does and of its need of funds. A footline in bold type announced that the expense of the advertisement was being defrayed by friends of the hospital; though the distinction between paying for it out of hospital funds and having it paid by generous friends direct from their own pockets is perhaps rather a fine one. At any rate, the page in question is an object-lesson in the fine art of charitable appeal; we have rarely seen a more telling or better designed advertisement of any kind. The novel way in which a large furnishing firm has been associated with the advertisement has probably reduced the cost which the

"friends of the hospital" have defrayed. The well-known ingenuity of the Hon. Sydney Holland has evidently not deserted him; and it is to be hoped the result of his quinquennial appeal will equal or surpass all records.

### AN INSURANCE CHART.

The intricacies of the National Health Insurance Act are sufficient to appal all those non-legal folk who are brought in contact with its ramifications, whether as employers, employed, doctors, society officials, or in any other way. With a view to simplification the managers of the *O. S. Review* (The Journal of the Organisation Society) have prepared a large Chart, wherewith the wayfarer may guide his path through the mazes of the labyrinth. Upon a large sheet, suitable for mounting upon a wall or a large door, the Act is analysed and synthesised upon a definite scheme, arranged so as to enable the searcher after information to find at once the clue to understanding. This sheet is published at one shilling, and must prove invaluable both to employers and employed. It does not contain an analysis of the conditions and regulations of medical benefit; for these, of course, are not laid down in the Act, but are drawn up by the Commission and administered by the Committees. A supplementary sheet containing these particulars would be useful, and it would not need to be so large. The labour of compiling this Chart must have been enormous; but then the organisation of such work is the *raison d'être* of the Society, and they are able to command the talent necessary for this particular kind of intellectual activity. The idea of the Chart is good, and its execution is at least equal to its conception. The result is a product which is a very good shillingsworth indeed.

### REPATRIATING ALIEN MENTAL PATIENTS.

Adopting the policy of repatriating alien mental patients where possible, the Asylums Committee of the London County Council have been able to arrange for a Chinaman, who since June 1911 had been a patient at Colney Hatch Mental Hospital, chargeable to the County of London, to be sent back to his native country. A certificate under the Aliens Act was obtained, but the Secretary of State did not see his way to make an expulsion order as the patient had served on a British ship. The Committee, however, were able to secure him an assisted passage to China, and the cost of the passage, £10, was defrayed in the first instance from a private fund at the Committee's disposal. Inasmuch as the expenditure has resulted in the relief of the county from further charges for the patient's maintenance, the Committee contend that the county should bear the cost of repatriation. Application has been made to the Local Government Board and they have sanctioned the expenditure.

### TREATMENT OF ACTON'S SCHOOL CHILDREN

Up to the passage into law of the National Insurance Act the Acton Urban District Council made a grant of £50 to the Acton Cottage Hospital in respect to the treatment of their workmen at the institution. With the passing of the contentious Act the Council intimated that they could no longer continue the grant in question, but desired to make an arrangement with the hospital under which payment should be made for the treatment of Council school children suffering from adenoids, enlarged tonsils, etc. Cordial relations have always existed between the Council and the hospital, and, as the result of a conference between delegates from the two bodies, the hospital executive have suggested the following terms for the Acton Council's consideration—viz., the hospital to enter into an agreement with the Council for treating the children at 7s. each, the local Education Committee paying in addition the doctor's fees for attending each case; or, alternatively, the hospital executive to enter into an agreement, say, for one year, to treat all cases for a fixed sum (£50 has been suggested), the Education Committee paying in addition the doctor's fees.

### MOTOR-CAR PATIENTS AND VOLUNTARY HOSPITALS.

The Lord Mayor of Manchester has been actively interesting himself in an endeavour to increase the sum raised in Manchester and Salford on Hospital Sunday for the voluntary hospitals. Presiding at the annual meeting of the Manchester and Salford Hospital for Skin Diseases, the Lord Mayor said he had heard of cases of people coming in motor-cars to the hospital to get the benefit of the institution and contributing nothing towards its upkeep. He expressed a strong opinion that such conduct was most improper, a view which we share. But what have the hospital authorities to say on the point? Have the managers of the Manchester Skin Hospital permitted patients arriving in motor-cars to receive free treatment at that institution? If so, on what grounds do they justify their inaction in not putting a stop to grave abuses of this kind? The motor-car population is continually increasing in numbers. It includes a large majority of people who sadly lack discipline and so make themselves deservedly unpopular up and down the country. It may be beyond the powers of the Royal Automobile Club to formulate and enforce a code of manners for the observance of motorists, but it is certainly the duty of hospital authorities everywhere to make it impossible for the abuses referred to by the Lord Mayor of Manchester to prevail at any hospital.

### SHOULD OUT-PATIENTS CONTRIBUTE?

Mr. Richard Kershaw, who for very many years successfully organised free contributions from out-patients with complete success, has had some interesting and useful letters in the *Daily Graphic* on the subject. He properly maintains that although we move slowly in the hospital world and old usages die hard, if the London Hospital and Guy's Hospital can, with advantage, institute a system of payment by patients, other hospitals can just as easily do so. Mr. Kershaw maintains that the better-to-do patients are invariably those sent by doctors, and these patients are desirous of giving a contribution. It is essential, where payments are received, that no distinction should be made in the treatment between the contributing and the free patient. Mr. Kershaw maintains that the best charity is that which is conducted on business lines, and for this reason a system of payment by patients according to their means should be encouraged in every charitable institution throughout the country.

### RECENT DEVELOPMENTS IN ANTI-PHTHISIS MEASURES.

We have heard a good deal lately of the courses of instruction on tuberculosis now being given at Metropolitan Consumption Hospitals. These institutions undoubtedly do well to bestir themselves, but, to be quite frank, learners at the present time can hardly regard them as the fountain-head of information. The important recent developments in anti-tuberculosis measures are, of course, three—namely, the sanatorium and the two kinds of dispensary, the tuberculosis dispensary and that mainly for the administration of tuberculin. Dr. Latham has pointed out that none of these derives in the slightest from the consumption hospital out-patient department. If it were not for Dr. Paterson's work at Frimley, could the London school point to any achievement warranting them to be considered just now as anything but communicative learners? And so it is not surprising to find that such courses are strictly practical. Probably their chief use as yet is to give the general practitioner, to whom falls in the very first place the selection of cases, a good chance of rubbing up his auscultatory and other powers of diagnosis. In Germany, however, as we see by the prospectus of a post-graduate course on tuberculosis at the Hamburg General Hospital, matters have gone further. There are lectures on subjects like the differential diagnosis of tuberculous diseases of the central nervous system and on the mental condition of consumptives; while, lest anyone should say that their ambition, like that of Gambado, is to add to the theory with as little practice as possible, those responsible also give disquisitions on the treatment of laryngeal phthisis and the choice of cases for admission to a sanatorium. Characteristically enough the lectures are published, and the first volume runs to 324 pages with 69 figures in the text. It must be confessed that the Teuton scientist seldom fails to outwork his rivals.

### HOSPITAL ACCOMMODATION FOR LEPROSY CASES.

At a meeting of the Metropolitan Asylums Board on February 8 the General Purposes Committee announced that a communication had been received from the Greenwich Borough Council stating that that body had made leprosy a notifiable disease, and asking the managers to express their willingness to provide hospital accommodation for the treatment of cases. The Committee recommended the Board to inform the Greenwich authorities that they were not prepared to endorse the view that special hospital accommodation was necessary for cases of leprosy, either in the interests of the lepers or of the public health. The Board's Medical Officer reported in support of the Committee's recommendation that, though the Local Government Board has knowledge of a number of lepers, there is only one instance on record of a previously healthy person developing the disease in these islands. While there is no doubt that leprosy is contagious, it is only so in a low degree, and prolonged and intimate contact are necessary to permit of the spread of infection. He considered that the lepers who could be maintained in their own homes with proper sanitary precautions might safely be left there, and that those living in unhygienic conditions could be treated in the London hospitals or infirmaries without risk to the other inmates. He added that the idea that leprosy is dangerously infectious is firmly rooted in the public mind, and is maintained by the deaths from leprosy which occur from time to time in the ranks of those who tend the sufferers from the disease. No account is taken, however, of the very large number who escape infection. The Board adopted the Committee's recommendation.

### THE TREATMENT OF TUBERCULOSIS IN LONDON.

An important report on the treatment of tuberculosis in London has been prepared by the Public Health Committee of the London County Council. The Public Health Committee point out that the work of the various authorities and institutions must be co-ordinated. Under the existing arrangements made by the Local Government Board, the provision of dispensaries is being dealt with by the sanitary authorities, but it is very important that the part played by the hospitals, general and special, should not be displaced by any newer dispensary provision. The dispensaries, if established as separate institutions, should fill up the gaps where the hospital provision is inadequate. It would be a great misfortune, the Committee add, if the hospitals, with their elaborate equipment and special organisation, were not utilised in any future arrangements. The Committee point out that at present the local authorities, hospitals, and, in fact, all concerned in the provision of treatment for tuberculosis in London find it difficult to decide what arrangements to make for the future treatment of the disease, because of the absence of any co-ordinating scheme. The Council's Medical Officer estimates that there are in London 50,000 persons suffering from tuberculosis, and of these about 20,000 are insured persons. Contributions towards the expenditure involved in the scheme will come both from the Government and the Insurance Committee, but until we know what relation these amounts will bear to the estimated cost its scope remains quite in the air.

### KING'S COLLEGE HOSPITAL OLD SITE.

In The Hospital of November 30 last it was stated that the site and buildings of the King's College Hospital might pass into the hands of the Metropolitan Water Board, which was desirous of securing new central offices. It does not seem, however, as if this project is likely to materialise, but as the result of the invitation to the public to tender for the building, an offer to acquire the site for commercial purposes has been made to the governors.

### THE ADVERTISEMENT OF STAFF VACANCIES.

A short time ago we felt constrained to point out that one of the general hospitals in London advertised a vacancy on the visiting staff in such a form as to make it appear that applications were not desired, and that free competition was to all intents a dead-letter. The announcement stated that a physicianship was vacant, but nothing was stated regarding the conditions of the post, the date of closing of entries, or any other of the usual details. Immediately following appeared a statement that a vacancy for an assistant physician existed, and full particulars were given of the qualifications required, testimonials to be submitted, dates, and other material matters. We felt obliged to point out that the covert intimation thus given that no one need apply for the senior post was a way of defeating the whole purpose of advertising it. It may be true that the assistant physician next on turn for promotion had such claims that no opposition to him was likely to be successful. But such an opinion is out of place in a formal notice of the vacancy. Were it generally adopted, promotion would be even more rigid and stereotyped in the voluntary hospitals' staffs than it already is. It very seldom happens that the senior assistant surgeon or physician fails to secure election to the full staff when a vacancy occurs; but it does happen sometimes, and it is not desirable that this possibility should be abolished. The method of advertisement to which we allude is, therefore, contrary to the spirit and object of the rules which most hospitals adopt as to the public advertisement of vacancies; and we are sorry to see the Brompton Hospital for Consumption now following the bad example recently set by the Middlesex. So similar are the formulæ which the two hospitals adopt that the inference of imitation seems inevitable. We trust the fashion will not spread any further, for it is a thoroughly bad one.

### DEATH OF TWO OPHTHALMOLOGISTS.

King's College, London, and King's College Hospital have suffered a notable loss through the death of Malcolm Macdonald McHardy, on February 8 at Dumfries, late Professor of Ophthalmology at King's College and senior ophthalmic surgeon to the hospital. Professor McHardy was of sailor ancestry, for his father was an admiral and his grandfather was Nelson's flag-lieutenant on the *Victory* at Trafalgar. Notwithstanding his Scottish names, his medical education was English. He was a student at St. George's Hospital when Mr. Brudenell Carter had established his reputation there as a teacher and an ophthalmic surgeon. Just at that time—the early 'seventies—Mr. Carter had two pupils of unusual promise in Messrs. Frost and McHardy. The former in due course obtained promotion at his own hospital and school; the latter had therefore to seek for an opportunity elsewhere. Not only King's College Hospital, but the Royal Eye Hospital and St. John's Hospital also were glad to avail themselves of his services, and he served them all for many years most loyally, retiring a comparatively short time ago. As a consulting surgeon for diseases of the eye Mr. McHardy attained great success; and his strongly marked individuality and personality stood him in good stead when dealing with his large circle of private patients. A long series of valuable researches stands also to his credit, though he left practically no enduring mark upon the systematised literature of ophthalmic surgery. On the same day died also Henry Eales, senior surgeon of the Birmingham and Midland Eye Hospital, and a contemporary in the medical profession of Professor McHardy. Mr. Eales was consulting surgeon to a large number of local hospitals in the Birmingham district, and was Middlemore lecturer in 1897. He was also President of the Ophthalmological Section of the British Medical Association in 1911.

### A LEGACY TO THE ASYLUMS BOARD.

At the meeting of the Metropolitan Asylums Board on February 8 the Finance Committee reported the facts regarding a curious legacy. Under the will of a Mrs. E. R. Johnson, deceased, three sums, amounting in all to £7,000, have become payable to three of the fever hospitals which the Board provides and manages. A discussion followed the presentation of this report, and it was suggested that the deceased lady must have imagined that the hospitals in question were charitable institutions supported by voluntary contributions, instead of by the rates. It was accordingly argued that the Board would be acting dishonestly if the money were put to the relief of the rates, and that the proper course to take would be to get powers to apply the money for the benefit of voluntary hospitals in London. A proposal was brought forward and strongly supported with this object in view; but was defeated in favour of a motion which practically defers any decision until the money has been received. The suggestion to divide the £7,000 among voluntary hospitals will naturally receive sympathetic attention from those who manage the latter institutions; and on many grounds it is a praiseworthy idea. The two difficulties which will probably prevent its adoption are: first, the legal objection to alienation of its rightfully acquired funds by any public rate-supported body; and, secondly, the squabbles that might ensue over the division of the fund. We cannot imagine any plan for solving this latter problem which would be equitable and also sufficiently popular to have a chance of being carried; but if any hospital secretary thinks he can, by all means let him communicate his views to us, and we will gladly publish them.

### AN ALTERNATIVE TO CLEAR WINDOWS.

There occurs in every institution, and particularly in those situated in large towns, a block of buildings giving anything but a cheerful prospect from its windows. This is rarely found in the wards themselves, but more usually in the staff-room block, in the nurses' or the sisters' sitting-rooms. The greatest care is taken to place the nurses' home as far from the operative buildings as possible, so that the staff may spend their leisure right away from the environment of the institution proper, and this is most desirable, unless the outlook from the windows is on to an ugly brick boundary wall. This not only shuts out the sunshine, but is in itself anything but inspiring. We came across an instance of this a few days ago. A room originally planned for a sitting-room was so unpopular on account of its dismal outlook that the energetic superintendent promptly converted it into a linen sorting room, in the hope possibly that if the eyes were engaged the depressing atmosphere would not be noticed. What a strange fascination clear glass has! We must have it free from specks, bubbles, or other blemishes, as the architect's specification says, and never pause to wonder why we are so fond of it. A room should not be dependent on its outlook for its charm; the charm should rather lie in the decoration and furnishing of the room itself, and why, therefore, have clear glass when the prospect is unpleasing? Would it not be better to glaze with an obscured glass which would break up and reflect the light, and give a well illuminated, cheerful chamber instead of one depressing and dismal? Substitutes for clear glass, whether merely translucent, as in the frosted varieties, or actually coloured, are worth attention.

### A QUESTION FOR THE BRADFORD CLERGY.

The representatives of the workpeople of Bradford on the Joint Hospital Fund (Incorporated) are very much dissatisfied, we are informed, with the clergy's response in Bradford to Hospital Sunday. There are 130 places of worship in the city where such collections are made, but the total received last year only amounted to £650, which was nearly the lowest amount that has been received. It is alleged that Church officers, more than any other body of people, are ever ready to take advantage of the recommendations issued in connection with the Hospital Fund, and on this ground it is felt that these officials ought to make greater efforts to increase the collections from places of worship. What have the Bradford clergy to say on the matter?

### MENTAL HOSPITALS' WORKMEN'S COTTAGES.

Two blocks, each consisting of three cottages, have been completed at Netherne County Mental Hospital. Each cottage contains a living room, parlour, and scullery, with larder, w.c., and coal placed on the ground floor, while three bedrooms are provided above. Being in an exposed situation on top of a steep hill the walls are built hollow, and the total cost, including cartage three miles from the nearest station, was £275 each cottage.

### TWO LECTURES ON HOSPITAL CONSTRUCTION.

We would draw the attention of our readers to lectures on Modern Hospitals at No. 9 Conduit Street, W., the premises of the Royal Institute of British Architects, on Monday, the 17th inst., at 8 p.m. The President will occupy the chair, and two papers will be read, one by Mr. A. Saxon Snell, F.R.I.B.A., the well-known hospital architect, and the other by Mr. William Milburn, A.R.I.B.A., whose papers on "Modern German Hospitals" have already been reviewed in our columns. Those interested in modern hospital planning will be cordially welcomed.

### A CURIOUS CASE OF MERCURIAL POISONING.

The circumstances attending the death of a child, which were revealed at an inquest recently held at Coventry, provide a useful lesson on the administration of drugs. The child suffered from ringworm, and the mother, after applying various remedies, consulted the family doctor, who supplied her with a solution of iodine, and gave her a prescription for ammoniated mercury ointment. The mother applied both the preparations to the child's head, with the result that the child suffered considerably, and died a few days afterwards from mercurial poisoning. The result of mixing the iodine solution and the mercurial ointment was the formation of the poisonous salt biniodide of mercury, which became absorbed. The doctor, in his evidence, said his instructions were that the iodine was to be painted on to the ringworm patch, and that on no account was the ointment to be put on the patch at the same time. The jury came to the conclusion that the mother had misunderstood the directions, but expressed the opinion that medical men should give more definite instructions as to the use of prescribed remedies.

### THE SALE OF SYNTHETIC HYPNOTICS.

The Council of the Pharmaceutical Society, at its February meeting, passed a resolution adding to Part II. of the Poisons Schedule diethyl-barbituric acid, a synthetic product which is known under several trade names, *e.g.* veronal. This resolution has been sent to the Privy Council, without whose approval it cannot take effect. If, and when, the Privy Council consents to the addition of this substance to the list of poisons its sale by retail will be restricted to registered pharmacists, who will be required to place a poison label on any package containing it. In view of the number of fatalities attributed to diethyl-barbituric acid it is certainly desirable that some precautions should surround its sale.

### THIS WEEK'S DRUG MARKET.

Business has been quieter during the week, and the improvement noticed a week or two ago has not been maintained; the outlook, however, is fairly good. Few changes in value of any note have taken place since last report appeared. Buyers of opium are holding aloof from the market, and quotations are unchanged. The excitement in essence of lemon has quietened down somewhat, and prices are if anything rather lower. Citric acid, however, is tending upwards in value. Sarsaparilla is slightly dearer. Reports concerning the cod-fishing in Norway continue to be favourable, but very little business is being done in cod-liver oil at present. The prices of borax and boric acid have been advanced.

